# Spatially resolved lipids in a mouse brain model of globoid cell leukodystrophy via IR-MALDESI MSI and parallel reaction monitoring MSI

**DOI:** 10.1007/s00216-026-06326-3

**Published:** 2026-01-23

**Authors:** Sierra N. Hunter, Mary F. Wang, Brittany N. Thomas, Anthony J. Filiano, David C. Muddiman

**Affiliations:** 1 Biological Imaging Laboratory for Disease and Exposure Research (BILDER), Department of Chemistry, North Carolina State University, Raleigh, NC 27695 USA; 2 Marcus Center for Cellular Cures, Duke University, Durham, NC 27705 USA; 3 Department of Neurosurgery, Duke University, Durham, NC 27705 USA; 4 Department of Pathology, Duke University, Durham, NC 27710 USA; 5 Department of Integrative Immunobiology, Duke University, Durham, NC 27705 USA

**Keywords:** IR-MALDESI, Mass spectrometry imaging, Globoid cell leukodystrophy, Psychosine

## Abstract

Globoid cell leukodystrophy (GLD) is a genetic neurodegenerative disease caused by mutations in galactosylceramide β-galactosidase (GALC) that results in the accumulation of the cytotoxic sphingolipid, psychosine. As psychosine is a biomarker specific to GLD, identifying the most afflicted regions of the nervous system can assist in better understanding the disease mechanism. Infrared matrix-assisted laser desorption electrospray ionization (IR-MALDESI) mass spectrometry imaging (MSI) and parallel reaction monitoring were utilized to elucidate the spatial distribution of the psychosine analyte and confirm the identity of the ion in a sagittal section of a GALC-deficient mouse brain. The presence of the psychosine was increased in specific anatomical regions of the brain responsible for the bodily functions that are impaired by GLD (cerebellum and brain stem). Several electrospray solvent additives (dopants) have enhanced the detection of various analyte types but with little success in enhancing the detection of sphingolipids. This study investigates the usefulness of ammonium fluoride electrospray doping in the positive ion mode for lipidomic IR-MALDESI MSI analysis.

**Graphical abstract:**

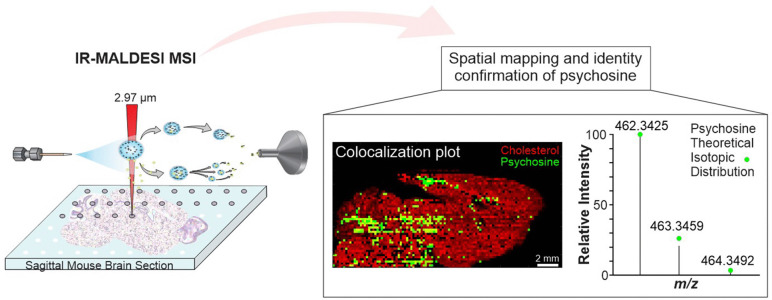

**Supplementary information:**

The online version contains supplementary material available at 10.1007/s00216-026-06326-3.

## Introduction

Mass spectrometry imaging (MSI) is a powerful analytical method that can determine the spatial distribution of hundreds to thousands of analytes in biological samples and visualize the morphological characteristics of the tissue specimen [1, 2]. By simultaneously observing the composition and the distribution of chemical species in biological samples, MSI provides crucial insight to the physiological mechanisms of tissue function [3]. Mapping the distribution of molecules in healthy tissue establishes a baseline from which alterations in diseased states can be identified. In the context of neurological disorders, the brain has been extensively researched, and MSI has provided many advancements in the understanding of Alzheimer’s disease [4], Parkinson’s disease [5], amyotrophic lateral sclerosis [6], and stroke models [7].

Globoid cell leukodystrophy (GLD) is a genetic neurodegenerative and demyelinating disease caused by a genetic deficiency, or total absence, of the lysosomal enzyme galactosylceramide β-galactosidase (GALC) [8–12]. This condition results in the accumulation of the cytotoxic sphingolipid, psychosine, which is pathognomonic to GLD making it an ideal biomarker for the disease and its progression [9–12]. Twitcher mice carry a similar GALC mutation, making them an ideal animal model to study GLD. Previous studies of GLD have primarily utilized non-tissue samples (e.g., blood-spots, lipid extracts) or have involved the homogenization of tissue for analysis using ESI-MS/MS or LC-MS [9–15]. Imaging of proteins on GLD tissue samples has been largely accomplished via immunohistochemistry and immunofluorescence; however, this leaves a rather large gap in the knowledge regarding the distribution of other analytes [12–14].

Infrared matrix-assisted laser desorption electrospray ionization (IR-MALDESI) is a hybrid MSI ionization technique combining benefits of matrix-assisted laser desorption ionization (MALDI) and electrospray ionization (ESI) to measure a variety of biomolecules [1]. The use of an IR laser enables the use of exogenous matrices that absorb at 2.97 µm including a thin exogenous ice layer [16], glycerol [17], and sucrose [18] that function as an energy-absorbing matrix which facilitates the desorption of neutral species into the orthogonal electrospray plume [16]. This research aims to utilize IR-MALDESI MSI to elucidate the relative abundance and location pattern of psychosine in an end-stage GLD Twitcher mouse brain model. Finally, we attempt to enhance lipid detection using a variety of chemical and instrumental methods previously reported.

Paraformaldehyde (PFA) fixation and sucrose-embedding, when paired together, were demonstrated to effectively maintain the integrity of biological tissues and prevent damage during the flash-freezing process, allowing for more concrete biological conclusions to be drawn from MSI data and tissue histology [19, 20]. In IR-MALDESI MSI analysis, sucrose-embedding tissues provide the additional benefit of the sucrose acting as a permeative mid-IR energy absorber. Previous lipidomic analysis of sucrose-embedded mouse brain tissues using IR-MALDESI MSI exhibited enhanced detection of lipids and better visualization of localization for specific targeted analytes than what was obtained with fresh-frozen mouse brains [21, 22]. These methods were implemented in this study to further metabolic and lipidomic coverage in these analyses.

In addition to these tissue preparatory methods of improving lipid detection and abundance, the localization and distribution of lower abundant molecules of interest, such as psychosine, may be further evaluated using parallel reaction monitoring (PRM) MSI. PRM is a method of tandem mass spectrometry (MS/MS) where all fragments of a targeted precursor are monitored at once, measuring the full MS/MS spectrum and thus providing a complete profile of the precursor ion.

ESI solvent compositions often vary depending on the method of mass spectrometry analysis and its optimization of ion formation based on what minimizes matrix effects and improves both sensitivity and reproducibility [23]. Additives, such as formic and acetic acid, have become a staple for enhancing ionization in ESI methods; however, more recently, there has been an expansion of research to other potential additives that may be used to further increase the sensitivity of particularly low-abundance ions or cause an abundant formation of a particular adduct to detect. Such investigations have included additives consisting of lithium, silver, ammonium, acetate, and fluoride [24–27].

Silver nitrate (AgNO_3_) was investigated as an additive to the IR-MALDESI ESI solvent to improve detection of lipids in positive ion mode for both a targeted and untargeted lipidomic analysis [26, 28]. These studies displayed the silver cations’ high affinity toward π orbitals, particularly those with multiple π bonds and bay regions. However, in the untargeted analysis, it was determined that silver preferentially interacted with lipids that did not contain a hard base. No improved detection via silver adduction was observed for glycerophospholipids or sphingolipids [28].

Ammonium fluoride (NH_4_F) has been utilized as an effective additive for enhancing analyte sensitivity across various ionization methods including IR-MALDESI MSI, specifically in negative ion mode [29]. Other ionization techniques have found varying results for signal improvement of analytes using NH_4_F in positive ion mode [27, 30, 31]. Lipids, due to their low gas-phase basicity, are not as dependent on gas-phase proton transfer(s) for signal enhancement as other analytes [31]. This makes it more likely that the signal is more affected by various adduct suppression or formation dynamics. NH_4_F is hypothesized to act as a sequestration agent and reduce the formation of sodium and potassium adducts to thus increase the formation of [M + H]^+^ or [M + NH_4_].^+^ ions [30].

Throughout this study, we demonstrate the utility of these methods to improve lipid and metabolite coverage using IR-MALDESI MSI. This study is the first application of MSI to elucidate the spatial localization of cytotoxic psychosine to further biological understanding of the role that psychosine plays in the progression of GLD to improve treatment strategies.

## Experimental

### Materials

LC-MS-grade water (H_2_O), acetonitrile (ACN), and formic acid (FA) for the electrospray solvent along with isopentane for flash-freezing were purchased from Fisher Scientific (Nazareth, PA, USA). Ammonium fluoride (NH_4_F) was purchased from Sigma Aldrich (St. Louis, MO, USA). Plain glass slides (1 mm height) were purchased from Fisher Scientific (Pittsburgh, PA, USA). Psychosine quantitative mass spectrometry standard was purchased from Avanti Research (Alabaster, AL, USA).

### Sample preparation

Three mouse brains were used for these measurements, and all experiments were performed according to the Institutional Animal Care and Use Committee (IACUC) at Duke University. Two mouse brains were PFA-fixed followed by sucrose treatment for cryoprotection. PFA-fixation was completed through transcardiac perfusion with phosphate-buffered saline (PBS, pH = 7.4) followed by 4% (w/v) paraformaldehyde. The extracted brains were stored in 30% sucrose solutions until the brains descended to the bottom of the jars [32]. The third mouse brain was fresh-frozen without any modification prior to flash-freezing. The fresh-frozen and sucrose-embedded brains were frozen after preparation, by floating the tissues over isopentane cooled by dry ice. Following flash-freezing, all brain samples were stored at −80 °C until time of analyses.

**Fig. 1:**
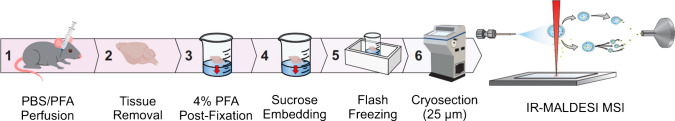
Experimental workflow for tissue preparation prior to IR-MALDESI MSI analysis

Sagittal tissue sections were collected from all mouse brains at 25 µm thickness at −20 °C with a Leica CM1950 cryostat (Buffalo Grove, IL, USA). The degeneration of the GLD model mouse brain led to the utilization of this thickness so as to obtain a whole tissue section without holes for imaging. Sagittal sections from the same biological tissue sample were used as technical replicates. In the sucrose-embedded brains, three replicates of the wild-type and four of the Twitcher brains were analyzed. Tissue sections were thaw-mounted on glass slides prior to IR-MALDESI-MSI analysis (Fig. 1).

### IR-MALDESI-MSI analysis

The IR-MALDESI source is coupled to an Orbitrap Exploris 240 mass spectrometer (Thermo Fisher Scientific, Bremen, Germany) as described previously [1]. For conditions where an ice matrix was used, samples were placed on the Peltier-cooled stage. The enclosure was purged with nitrogen gas for approximately 5 min, and the stage was cooled to −8 °C and stabilized for 10 min. After ambient humidity was reduced and the sample cooled, the enclosure was reopened to form a thin ice layer on top of the sample. Once the ice layer was formed, the enclosure was closed and re-purged with nitrogen to control the humidity prior to MSI analysis. Where conditions did not require an ice matrix, these steps were omitted.

### IR-MALDESI MSI of psychosine

A 2970 nm mid-IR laser (JGMA, Burlington, MA, USA) was used for sample desorption, delivering 1.3 mJ/burst at a rate of 10 kHz with a spatial resolution of 100 µm. This spatial resolution was selected to observe key anatomical features of the brain for greater confidence in determining localization of the analytes while reducing overall experiment and data processing time. Other replicates, where the laser delivered on average 1.7 mJ/burst, were collected with higher spatial resolutions, 140 and 150 µm (minor difference attributed to inter-day acquisition variability), to further reduce experiment and processing time. The variation in spatial resolution between the technical replicates allowed for evaluation of which acquisition parameters may be most beneficial for future studies. Experiments were conducted in positive-ion mode, where orthogonal electrospray plumes were comprised of 50:50 water/acetonitrile (v/v) modified with 0.2% formic acid. [23] Mass spectra were recorded at *m/z* 200–800 and *m/*z 200–1000 in different replicates, where automatic gain control was disabled, and the injection time was fixed at 15 ms. [33] Ion abundances were measured in units of charges per second which refers to the total charges divided by the injection time. The EASY-IC was enabled during analysis to ensure high mass measurement accuracy (within ± 2.5 ppm) using fluoranthene as the lock mass ([M^•+^] *m/z* 202.0777). The mass resolution was set to 240,000_FWHM_ at *m/z* 200, the multi-RF injection threshold was set to four, and the S-lens RF-level was fixed at 70% for analyses. Parallel reaction monitoring (PRM) imaging of psychosine was recorded over *m/z* 40–500 where the precursor *m/z* was set to 462.3425, the charge state was 1, and the isolation width was 5 m*/z*.

### Ammonium fluoride ESI solvent additive

A 2970 nm mid-IR laser (JGMA, Burlington, MA, USA) was used for sample desorption, delivering 2.2 mJ/burst at a rate of 10 kHz with a spatial resolution of 150 µm. This spatial resolution was selected for the objective of assessing analyte abundance across the samples rather than focusing on spatial details of the brain while further reducing overall experiment and data processing time. Experiments were conducted in positive-ion mode, where orthogonal electrospray plumes were comprised of 50:50 water/acetonitrile (v/v) modified with 0.2% formic acid (1.6 µL/min at 3300 V) [23] and of 50:50 water/acetonitrile (v/v) modified with 0.2% formic acid and 70 µM NH_4_F (3 µL/min at 3300 V) [29]. Mass spectra were recorded at *m/z* 200–1000 to encompass a larger range of lipids and include more in the upper *m/z* range with all other parameters kept the same as described above.

### Data analysis

All.RAW data files were converted to.mzML files using MSConvert [34] and then to.imzML files using an imzMLconverter [35] prior to analysis in MSiReader v3.08 (MSI Software Solutions, Raleigh, NC) [36, 37]. The resulting imzML files were uploaded online to METASPACE for MSI metabolite annotation according to the LIPID MAPS Structure Database (LMSD) and evaluated based on their metabolite-signal match (MSM) score [38]. The false discovery rate (FDR) was set at 10% to widen the scope of total putative lipid annotations. Furthermore, all reported on-tissue putative identifications were categorized into lipid categories by utilizing the LMSD. The eight lipid categories are Fatty Acyls [FA], Sterol Lipids [ST], Sphingolipids [SP], Glycerolipids [GL], Glycerophospholipids [GP], Polyketides [PK], Prenol Lipids [PR], and Saccharolipids [SL]. For consistency across samples in the evaluation of IR-MALDESI MSI of psychosine, data from all replicates was evaluated only in the *m/z* 200–800 range [38–40]. 

**Fig. 2:**
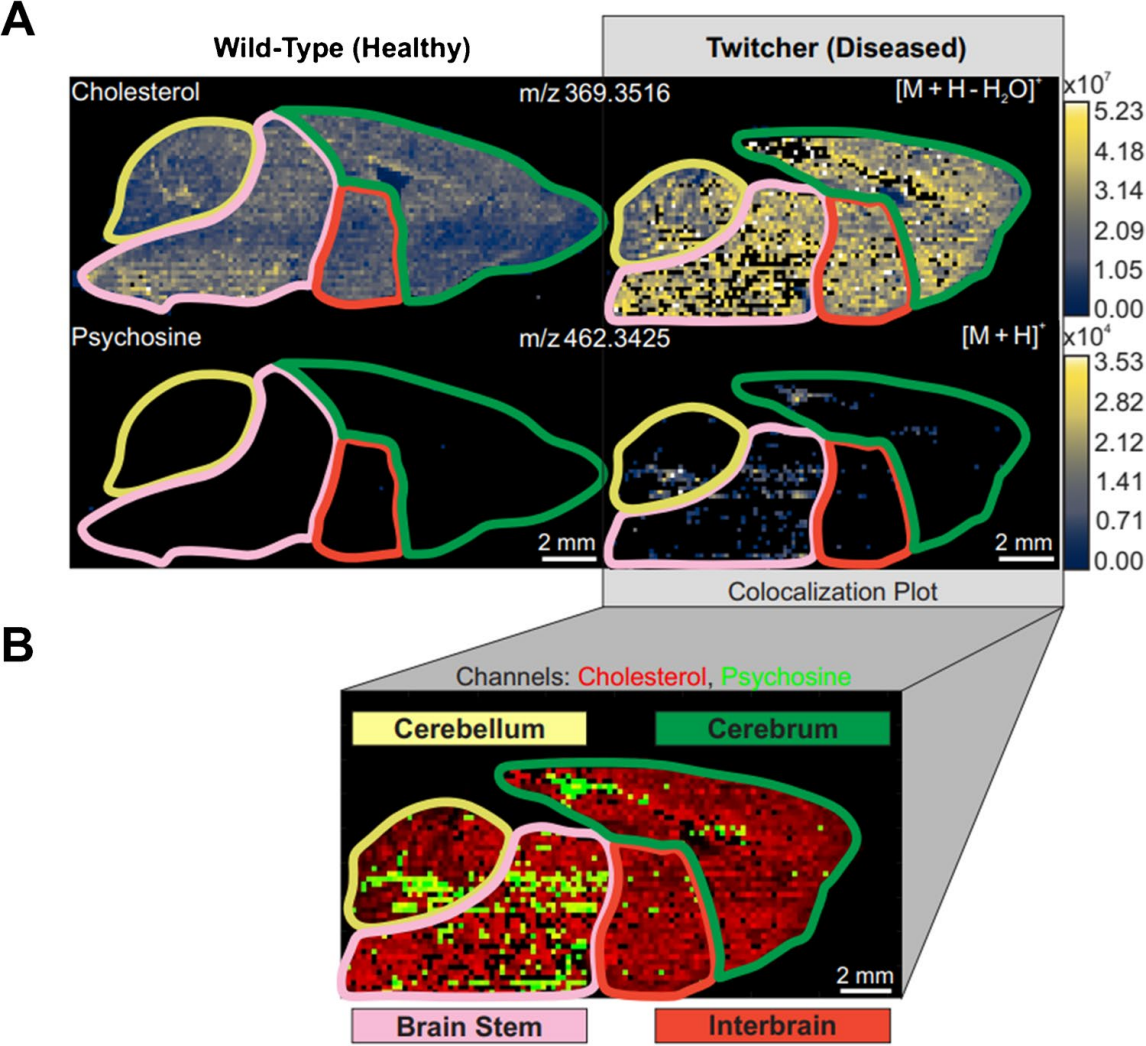
**A** Ion heat maps of cholesterol and psychosine for the wild-type and Twitcher mouse brain with the quadrants drawn in. **B** Colocalization plot of cholesterol and psychosine in the Twitcher mouse brain

To evaluate the spatial heterogeneity of lipids across replicates, square bioinformatic regions of interest (ROIs) (*n* = 40) were used for data extraction within each tissue section. The brain was partitioned into quadrants (Fig. 2, Figure S1) based on spatial similarity, and 10 ROIs were extracted from each region. The *n* = 10 of each region was used as pseudo-technical replicates for the measure of variability across that region. As the brain is a heavily heterogeneous tissue, concerns arise as bioinformatic ROI analysis inherently assumes spatial homogeneity within a sample; however, the in-sample biological variation can be reflected in the comparison of the ROIs across all four brain regions (*n* = 40). The variability across technical replicates of the same brain tissue was also investigated based on the ROIs’ extracted lipid abundances.

## Results and discussion

In the single-blind IR-MALDESI MSI analysis, the two sucrose-embedded brain samples—one healthy (wild-type, WT) and one diseased (Twitcher)—were successfully distinguished. Several observations of the intact brains were made prior to experimentation that aided in preliminary identification. These include the structural deterioration of the mouse brain while sectioning the Twitcher (diseased) mouse brain, which drove the need for thicker tissue sections. Thicker sections were collected to maintain the integrity of the individual samples as the degeneration of the diseased brain caused gaps in thinner sections which would negatively impact the images collected and the derivation of any biologically relevant conclusions based on the localization of disease-relevant analytes. Beyond the difference in physical attributes between the brains, the detection of psychosine in the Twitcher brain and its relative scarcity in the WT brain provided further evidence for differentiating the disease (Twitcher) and healthy tissues.

In positive ion mode, with sucrose-embedding and an exogenous ice matrix, the IR-MALDESI source was able to detect the [M + H]^+^ of psychosine (*m/z* 462.3425), with a mass measurement accuracy (MMA) of 1.5 ppm. At the spatial resolution of 100 µm, this analyte displayed localization (Fig. 2A) primarily in the cerebellum and brain stem of the sagittal brain section. The psychosine abundance in the cerebrum region of the brain was also highly concentrated in the visual cortex.

When considering the known symptoms of GLD, this localization pattern of psychosine aligns with the regions of the brain that would be responsible for causing those symptoms. The psychosine presence in the cerebellum, which controls muscular movement and balance, correlates to the GLD symptoms of muscle weakness and spasticity (Supplemental Figure S1) [8].

As expected, larger spatial resolutions resulted in some loss of visualizing distinguishable anatomical features in the brain sections. Thus, the localization of specific analytes such as psychosine was not as exact as that which was observed with the smaller spatial resolution (Supplemental Figure S2). However, throughout the technical replicates of the Twitcher mouse brain, regardless of spatial resolution, an accumulation of psychosine in the posterior regions of the brain (brain stem and cerebellum) is observed. The replicates of the wild-type mouse brain, in comparison, reveal no consistent abundance across any region of the tissue which is in line with what would be expected in a model with functional GALC enzymes.

Beyond determining the localization of psychosine, the colocalization plot of cholesterol and psychosine at the more detailed 100 µm resolution (Fig. 2B) reveals an interesting trend between these two analytes. While there is visible overlap (yellow pixels) of the two ions, a large portion of the detected psychosine is present where there are gaps in the cholesterol which may indicate that psychosine, or some byproduct of GLD caused by psychosine toxicity, interferes with the brain’s cholesterol metabolism. Recently, Bai et al. produced a review that focused on the relationship between cholesterol levels in the brain and the neurodegenerative Alzheimer’s disease (AD) [41]. Several studies have observed region-specific fluctuations of cholesterol levels and have proposed multiple conceivable reasons as to why these fluctuations may occur including the reduction of: de novo cholesterol synthesis, cholesterol trafficking, or cholesterol catabolism [42–45]. While there is no known direct link between psychosine and the synthesis or distribution of cholesterol—the data presented in this work recognizes there could be a connection. Psychosine induces oxidative stress and inflammation in the brain which may then interfere with the proper functioning of proteins/enzymes responsible for forming or dispensing cholesterol, leading to the anti-correlation observed between the two lipids [9–12]. While this trend is not observed in the other replicates of the diseased tissue (Supplemental Figure S2), the larger spatial resolution results in a “smoothed out” image that does not present these types of small-scale variations in signal.

**Fig. 3:**
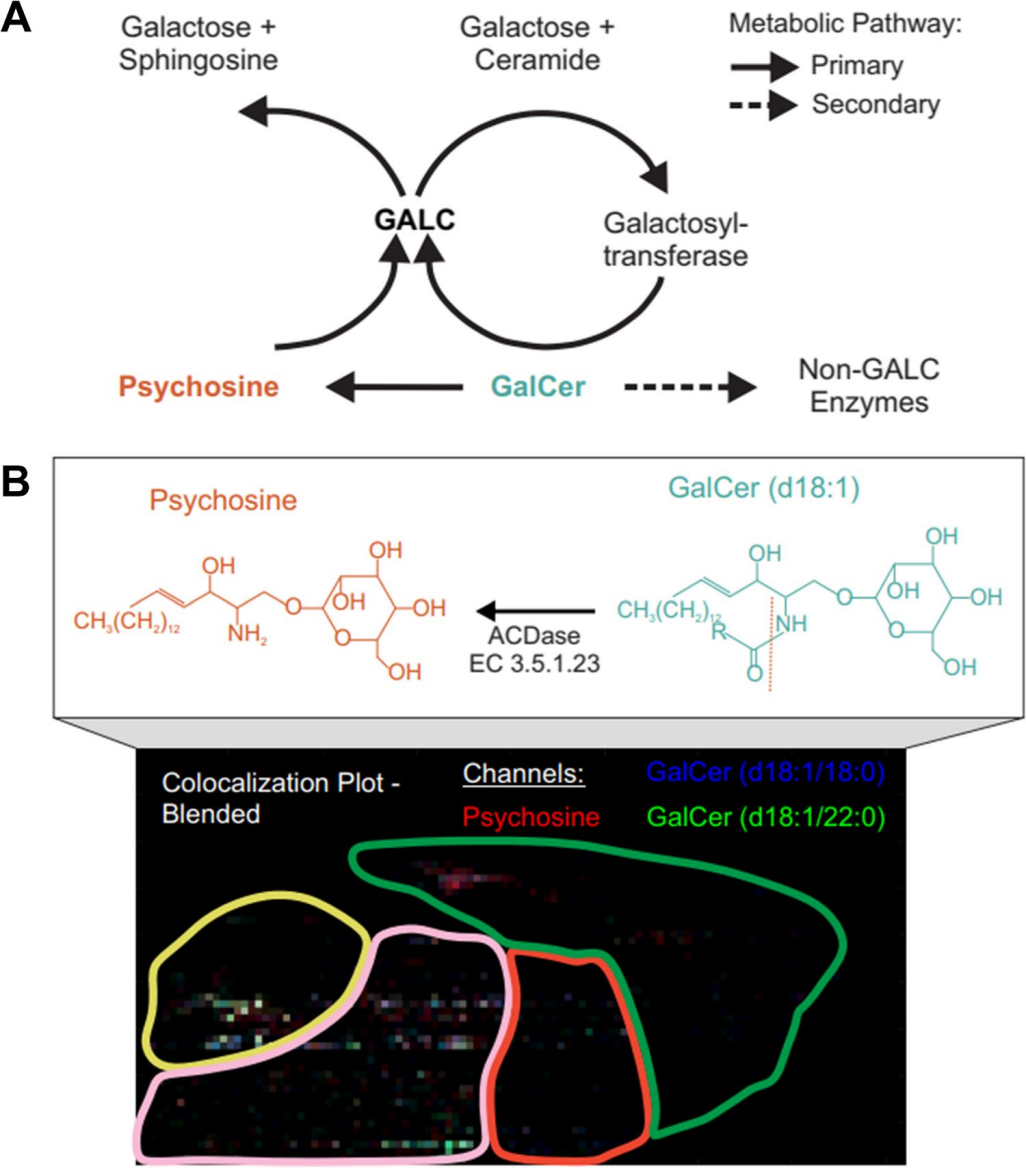
**A** Metabolic pathway of the GALC enzyme interacting with the GalCer and psychosine lipids whose accumulation is associated with GLD. **B** Colocalization plot of two highly abundant GalCer (d18:1) analytes in relation to psychosine. Channels are normalized separately, and the plot is blended so that where overlap of the *m/z* occurs, the colors mix

Further investigation detected several GalCer analytes that were upregulated in the Twitcher brain; this is consistent with the absence of the GALC enzyme that is responsible for the lysing of these lipids. In the formation of psychosine, Fig. 3A, the generally accepted pathway involves the catabolic deacylation of GalCer via acid ceramidase (EC 3.5.1.23) [12]. GalCer has two carbon chains, a sphingoid backbone and an acid chain, that can change in length and degrees of unsaturation. From those that fall into the *m/z* range of this dataset, the GalCer analytes that had a sphingosine chain of 18 carbons and one double bond (d18:1) localized to the same regions as the psychosine analyte (Fig. 3B, Supplemental Figure S3). A plausible conclusion based on these data is that of the GalCer lipid class, the d18:1 are the analytes responsible for forming the cytotoxic psychosine.

Bioinformatic ROIs were used to assess the regional differences in the ion abundances across replicates of both the WT and Twitcher mouse brains and determine whether these fluctuations were statistically significant. *Z*-scores of log-transformed abundances for each putatively annotated *m/*z were normalized by replicate, global, and region-global *z*-scores for evaluation in the form of boxplots (Supplemental Figure S4). Each normalization method was applied to the WT replicates and Twitcher replicates separately and into four subdivisions which represent the primary regions of the sagittal brain section (Supplemental Figure S4).

Although the brain is structurally heterogeneous, most of the investigated analytes exhibited consistent relative abundances across the four brain regions. This is indicated in Supplemental Figure S4A, which represents analyte abundances normalized to each replicate’s average of that analyte, as each boxplot mean across both tissue types and all brain regions falls within the range of ± 0.5 standard deviations. Global-normalized z-scores observe the analyte abundance in one brain region of one replicate regarding the average abundance of that analyte across all replicates of that tissue condition. Reflecting on this (Supplemental Figure S4B), an interesting trend is observed where there is higher consistency (*z*-score mean near zero) of abundances between WT replicates and more variability between the Twitcher replicates. However, by observing the analyte abundance in one brain region of one replicate regarding the analyte’s average abundance in that brain region across all replicates of that tissue condition (region-global normalization), the same replicates that had higher mean values in the global normalization also showed elevated region-global means across all four brain regions (Supplemental Figure S4C). This suggests a systematic offset of the abundance of those replicates rather than a biological or region-specific cause. The means falling within ± 0.5 standard deviations across all regions in every replicate indicates an expected range of variation between replicates of a complex sample.

**Fig. 4:**
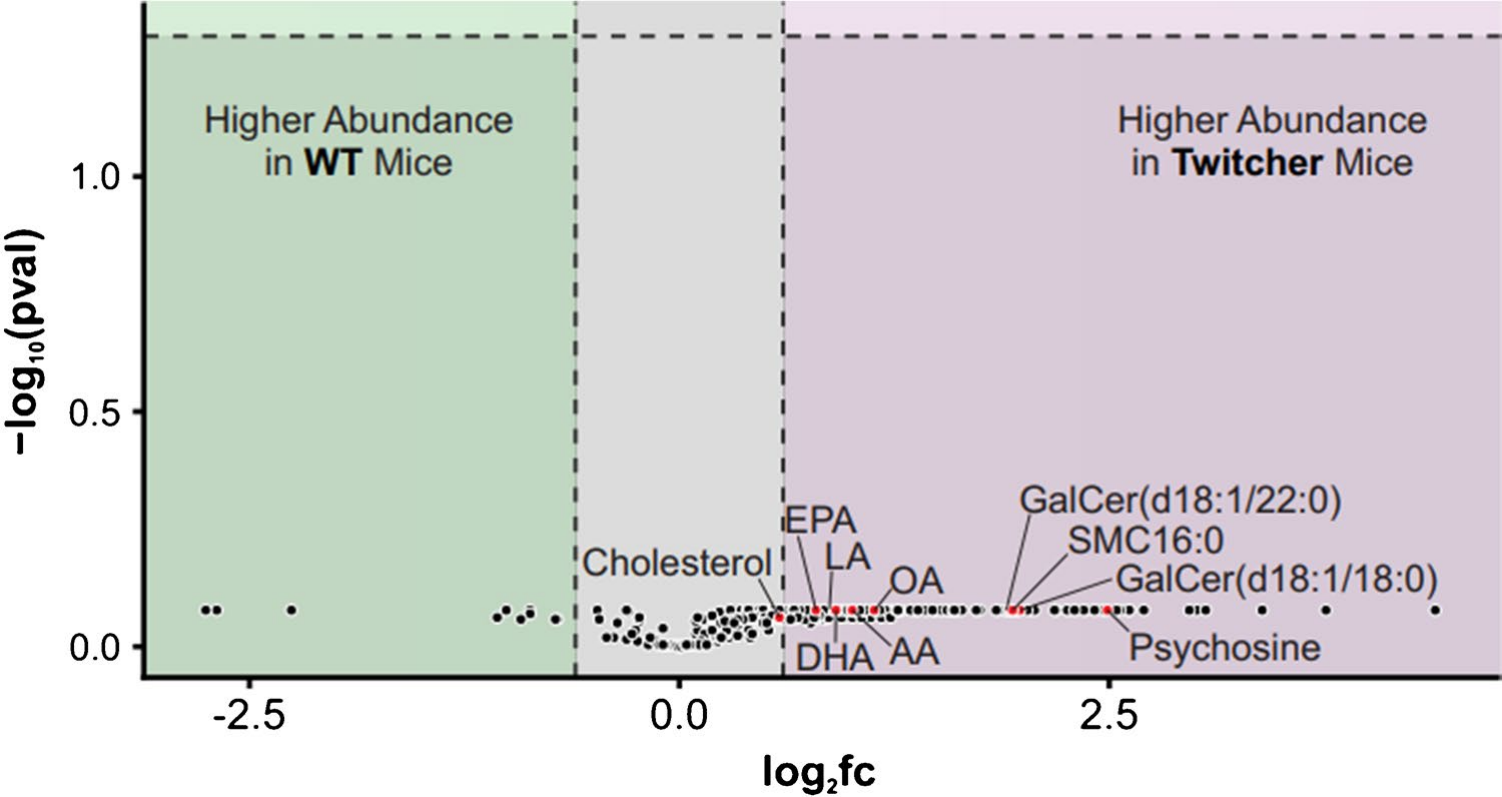
Volcano plot displaying log_2_fc versus -log_10_(adjusted *p*-value). Significance was determined using thresholds of |log_2_fc|> 0.6 and *p* < 0.05 after Benjamini-Hochberg correction [47]

Using the extracted abundances of the whole tissues of the technical replicates (*n* = 3 WT and 4 Twitcher), a volcano plot was produced to display the fold change of lipid abundance between the WT and Twitcher models (Fig. 4**)**. Lipids with a large effect size are the primary targets for disease biomarkers as they exhibit a pronounced difference in abundances between conditions. So, although no lipids analyzed met the threshold for statistical significance (*p* < 0.05), likely due to limited statistical power from the small number of replicates, several exhibited substantial effect size with the majority expressing a significant (|log_2_fc > 0.6|) increase in abundance in the Twitcher brain. This fold-change trend is similarly seen in other disease states where the upregulation of lipid abundances in the diseased state may be resultant of both a direct response and an indirect byproduct of the disease [7, 41, 46]. Directly, both pro-inflammatory and anti-inflammatory lipids accumulate in response to the disease states, reflecting active engagement of lipid-mediated immune regulation. In parallel, lipids not traditionally linked to inflammation also show altered abundance, likely due to secondary dysregulation of lipid metabolism driven by these disease processes. Several lipids that can be linked directly to an inflammatory immune response include oleic acid (OA), docosahexaenoic acid (DHA), eicosapentaenoic acid (EPA), linoleic acid (LA), and arachidonic acid (AA) which are all essential fatty acids and abundant throughout the entirety of the brain [46]. Considering the LMSD annotations, all five of these lipids exhibit a small to moderate effect size (0.6 <|log_2_fc|< 1.5) which may be the result of a more homogenous distribution that these lipids exhibit across the brain and the change in abundance between healthy and diseased tissue being consistent across the entirety of the tissues. Opposingly, psychosine has a very large effect size (|log2fc|> 2.0) which is due to the substantial difference in lipid abundances between the healthy and diseased tissues as well as the region-specific distribution of the analyte.

Of the lipids that have a biologically significant downregulation in the Twitcher brain, several are involved in the production of the five fatty acids linked to the inflammatory immune response [38–40, 46]. As for the lipids upregulated in the Twitcher brain, besides psychosine, GalCer (d18:1/18:0) and GalCer (d18:1/22:0) have relatively large log_2_fc values indicating that their upregulation in the diseased state is of biological significance which further validates the theory of GalCer (d18:1) compounds being precursors to psychosine.

Also seen with a large effect size and upregulated in the Twitcher mouse brain is an analyte of the sphingomyelin species, SM (d18:1/16:0) (SMC16:0). Sphingomyelins are typically located in cellular membranes and can be found in most tissue types [48]. However, in healthy brain tissue of both rats and mice, it has been recorded that longer fatty acyl-chain SM’s have higher concentrations while smaller-chained SM’s, like SMC16:0, are not very abundant [49, 50]. SMC16:0 does, conversely, make up a large portion (> 25%) of the sphingomyelin species in human serum [51].

A study conducted on diabetic rats observed alterations in sphingolipid composition of the brain including a significant upregulation of SMC16:0 in the prefrontal cortex and cerebellum [49]. Cognitive dysfunction has been associated with the different types of diabetes, the pathophysiology of which is uncertain; however, diabetes has been shown to cause neuroinflammation [52]. Inflammation is a key driving factor of blood-brain barrier (BBB) disruption and breakdown; the BBB is responsible for maintaining homeostasis by strictly regulating the movement of compounds between the blood and brain [53]. In a situation where the BBB is degraded and the serum starts to infiltrate the brain, an upregulation of SMC16:0 which is highly abundant in the serum, is plausible.

It is possible that the accumulation of SMC16:0 in the GLD brain is therefore indicative of BBB dysregulation. Slight BBB disruption has been observed in regular aging; however, exacerbated damage to the BBB has been linked to several other neurodegenerative diseases such as Alzheimer’s and Parkinson’s [53]. Other research has suggested that degradation of the BBB is involved in the progression of GLD but less so than observed in other dystrophies [54, 55]. As the BBB breaks down, more toxins and pathogens can infiltrate into the brain and cause neurological damage which may then aid in the progression of the disease and its symptoms.

As discussed above, many disease states cause lipid dysregulation with several neurodegenerative diseases observing similar trends of up- or downregulation. So, while this data presents several lipids whose dysregulation could be used as biomarkers for GLD, psychosine remains the primary focus as a pathognomonic marker of the disease.

Supplemental Figure S5 displays the volcano plots investigating the change in lipid abundances between tissue types within the four brain regions previously specified. Each regional-specific volcano plot was thus formed by ROIs collected in that region across replicates of the WT (*n* = 3) and Twitcher (*n* = 4) mouse brains. The most prominent thing to note in these figures is the lower, but still significant, effect size of psychosine in the cerebrum (**S5C**). This is likely due to the prominent localization of the analyte in the visual areas of the brain, as depicted in the Allen Mouse Brain Sagittal Atlas [56]. The visual areas of the brain make up a small area of the cerebrum as a whole, which contributes to the extracted abundance data reflecting a less significant upregulation than in the other, more prominently impacted regions of the brain.

**Fig. 5:**
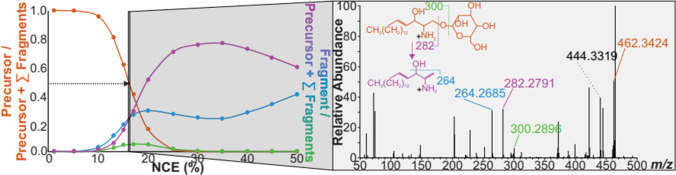
Breakdown curve optimizing collision energy for the psychosine standard on WT fresh-frozen mouse brain tissue. The fragmentation pathway of the quantitative fragments of psychosine is presented in tandem with the mass spectrum that depicts what the fragmentation pattern of psychosine looks like at 16% NCE

**Fig. 6:**
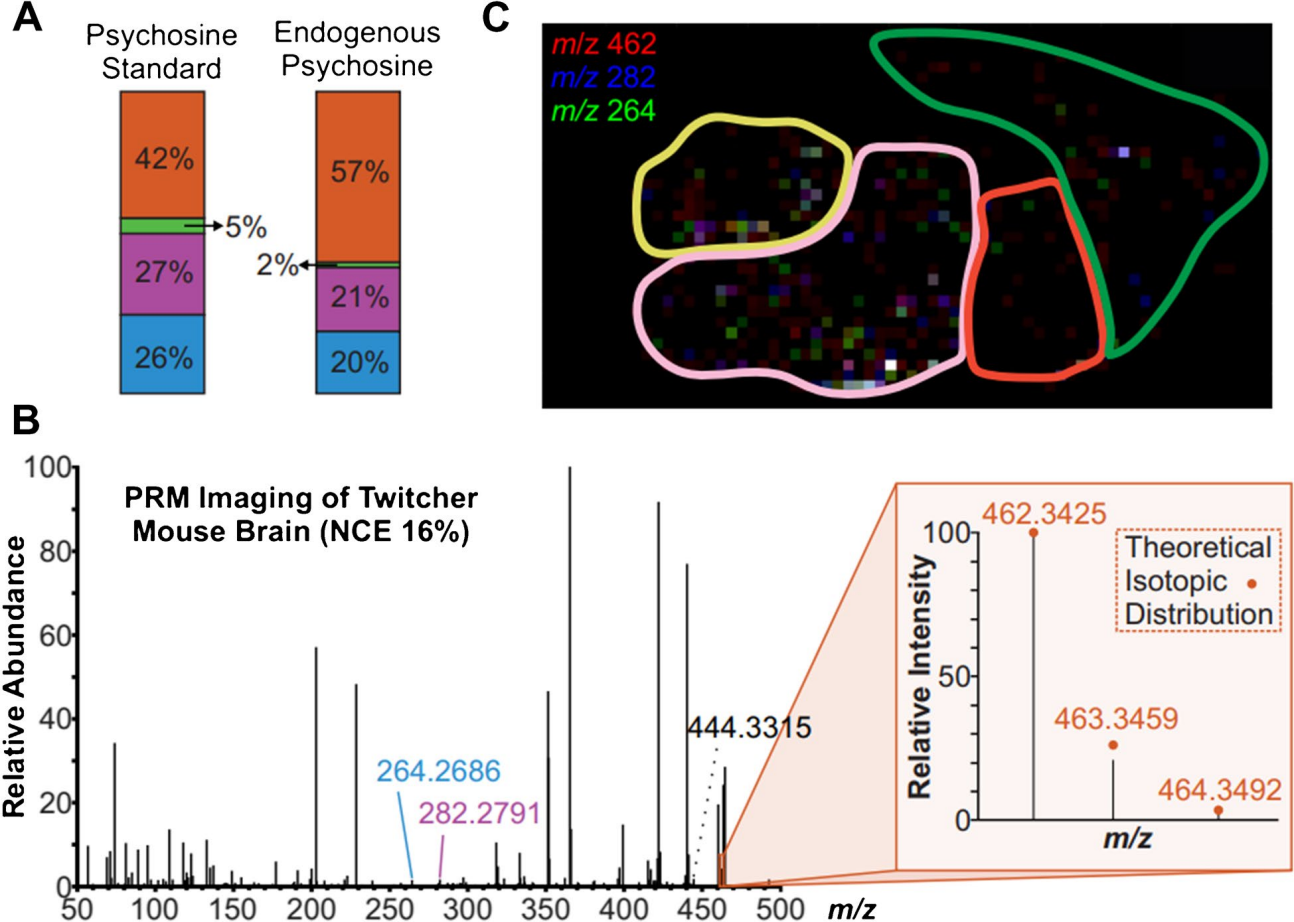
**A** Ion abundance ratio analysis at 16% NCE for the psychosine standard on a wild-type mouse brain and endogenous psychosine in a Twitcher mouse brain. **B** Mass spectrum and isotopic distribution of the PRM analysis of a Twitcher mouse brain section. **C** Colocalization plot of psychosine and its two abundant quantitative fragments

In addition to the presented data being consistent with known biological and metabolic pathways of psychosine, the fragmentation pattern of a psychosine standard was collected using tandem MS to confirm the observed *m/z* 462.3425 as the [M + H]^+^ peak for psychosine. In Fig. 5, the normalized collision energy (NCE) was optimized for the fragmentation of the standard at 16%. Three distinct quantitative fragment peaks were identified with two being particularly abundant (*m/z* 264 and 282). While both fragments are produced by a neutral loss of a galactose, the *m/z* 264 also loses a water molecule from the protonated psychosine ion. This may seem as though *m/z* 264 is a secondary fragment of 282; however, observing the breakdown curve (> 35% NCE), the larger fragments’ abundance starts to decrease in synchronization with the increase of the smaller fragments’ abundance. Therefore, the 264 peak is formed independently of peak 282 at NCE below 35% and can be treated as a quantitative ion rather than a qualitative ion. An ion abundance ratio analysis of the precursor and quantitative fragments of the pure psychosine standard showed that at 16% NCE, the 264 and 282 fragments should be approximately equivalent in abundance (Fig. 6A).

Parallel reaction monitoring (PRM) MSI targets a specific analyte for precise identification and quantification. Therefore, the isolation of the precursor ion (*m/z* 462.3425) and its subsequent fragmentation in the HCD cell at the optimized NCE for the psychosine standard confirms the identity of the endogenous psychosine within an MMA of ± 1.5 ppm. The mass spectrum produced from the PRM imaging, and the ion ratio analysis of the endogenous compound (Fig. 6A, B) show the quantitative fragments (*m/z* 264 and 282) as relatively equal in abundance, which is as expected according to the psychosine standard results. The change in the ion ratio analysis between the pure standard and the endogenous psychosine could be due to the wide isolation window used during PRM analysis (± 2.5 ppm) that may cause the precursor peak to co-isolate with other analytes.

The isotopic distribution of the *m/z* 462.3425 precursor peak aligns well with the theoretical isotopic distribution for psychosine (Fig. 6B). Additionally, the PRM images depict similar spatial distribution between the ion heat maps of *m/z* 264, 282, and 462 (Fig. 6C) which indicate that the *m/z*’s that match the quantitative peaks are indeed fragments of the psychosine precursor. All evidence tied together draws the conclusion that IR-MALDESI MSI was able to detect and localize where psychosine is most abundant in a late-stage Twitcher mouse brain.

While detected, psychosine is a relatively low abundance lipid (Fig. 6B). Various methods have been optimized to enhance lipid detection with the IR-MALDESI system in both positive and negative ion modes but with little success in enhancing sphingolipids. Recently, the NH_4_F concentration was optimized for lipid (70 µM) and glycan (350 µM) detection on the IR-MALDESI system; this resulted in up to a twofold and fourfold enhancement in ion abundance, respectively, in the negative ion mode [29]. The potential for NH_4_F signal enhancement in positive ion mode for IR-MALDESI MSI lipidomic analysis was explored with a focus on sphingolipids and determined to be less effective than in the negative ion mode.

Lipidomics by IR-MALDESI MSI of a sucrose-embedded Twitcher mouse brain were performed in positive ionization mode from *m/z* 200–1000 using both standard ESI (50:50 H_2_O: ACN, 0.2% formic acid) and NH_4_F doped ESI (50:50 H_2_O: ACN, 0.2% formic acid, 70 µM NH_4_F) [23, 29]. Two tissue samples were tested with each ESI solvent: one that used an ice matrix and one that used no ice matrix (four preparation methods total).

**Fig. 7:**
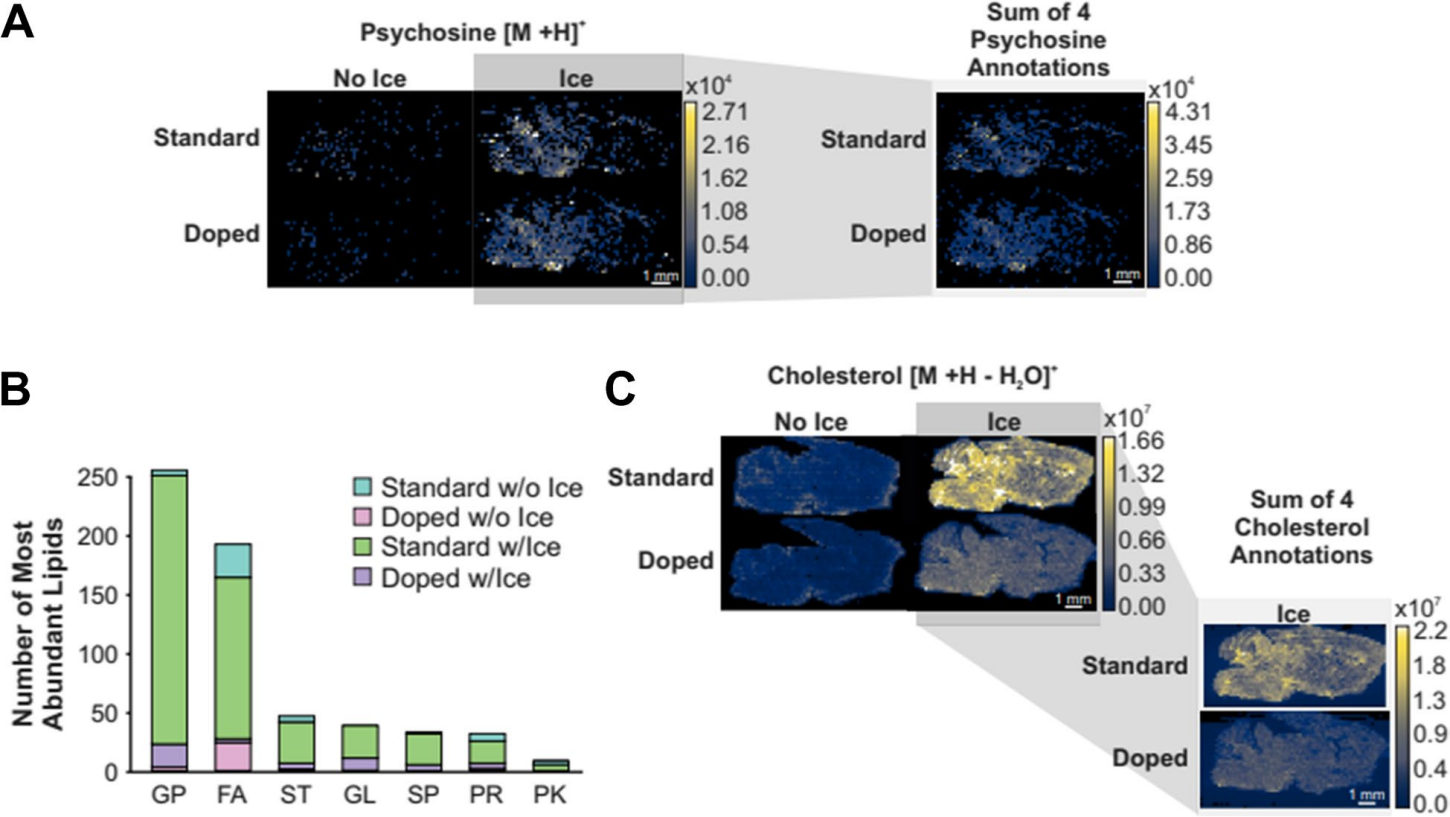
**A** Ion heat maps of psychosine (*m/z* 462.3425) across all solvent replicates and the summed ion abundance heat maps for the two ice matrix replicates. The four annotations are psychosine adducts: [M + H]^+^ (*m/z* 462.3425), [M + H-H_2_O]^+^ (*m/z* 444.3320), [M + NH_4_]^+^ (*m/z* 479.3691), and [M + Na]^+^ (*m/z* 484.3245). **B** Bar chart depicting the number of putative on-tissue lipids, based on LMSD annotations, that each treatment method had the highest abundance for. **C** Ion heat maps of cholesterol (*m/z* 369.3516) and the summed ion abundance heat maps for the two ice matrix replicates. The four annotations are cholesterol adducts: [M + H-H_2_O]^+^ (*m/z* 369.3516), [M + H]^+^ (*m/z* 387.3621), [M + NH_4_]^+^(*m/z* 404.3887), and [M + Na].^+^ (*m/z* 409.3441)

In Fig. 7A, the ion heat maps of psychosine (*m/z* 462.3425) are displayed for each of the four preparatory techniques. It is apparent that the application of an ice matrix enhances the detection of this sphingolipid in positive ion mode regardless of the ESI solvent composition. The abundances of the [M + H]^+^ psychosine analyte using the standard ESI solvent and NH_4_F doped solvent were not significantly different. However, given that the solvent additive contains ammonium, the potential for enhancement of alternate adducts rather than the typical ionization was evaluated. The “summed ion images” tool in MSiReader was used to sum the abundances of four psychosine adducts and combine them into one resulting ion heat map to compare overall detection of psychosine between the standard and doped solvents that were analyzed with an ice matrix applied (Fig. 7A). The summed ion heat maps express no significant difference in psychosine abundance when summing the four potential adducts.

The abundance of the on-tissue lipids was exported and analyzed based on their LMSD annotations to determine whether certain lipid categories responded better than others to the doped solvent (Fig. 7B). Of the sphingolipids detected, most expressed highest abundances with the standard solvent applied. Across the six other lipid categories detected in the mouse brains, the standard solvent with the use of an ice matrix produced the highest abundance for all types. This indicates that NH_4_F doping did not enhance detection of lipids in positive ion mode of IR-MALDESI MSI analysis.

To further establish the difference in detection between the two ESI solvent compositions, the ion heat maps of cholesterol (*m/*z 369.3516), a highly abundant and distinguished analyte, are displayed for each of the four preparatory techniques (Fig. 7C). The importance of the ice matrix in enhancing detection is further exhibited, and the summed ion heat maps of four potential adducts show that rather than enhancing the signal, NH_4_F doping had a dampening effect on cholesterol ion abundance.

**Fig. 8:**
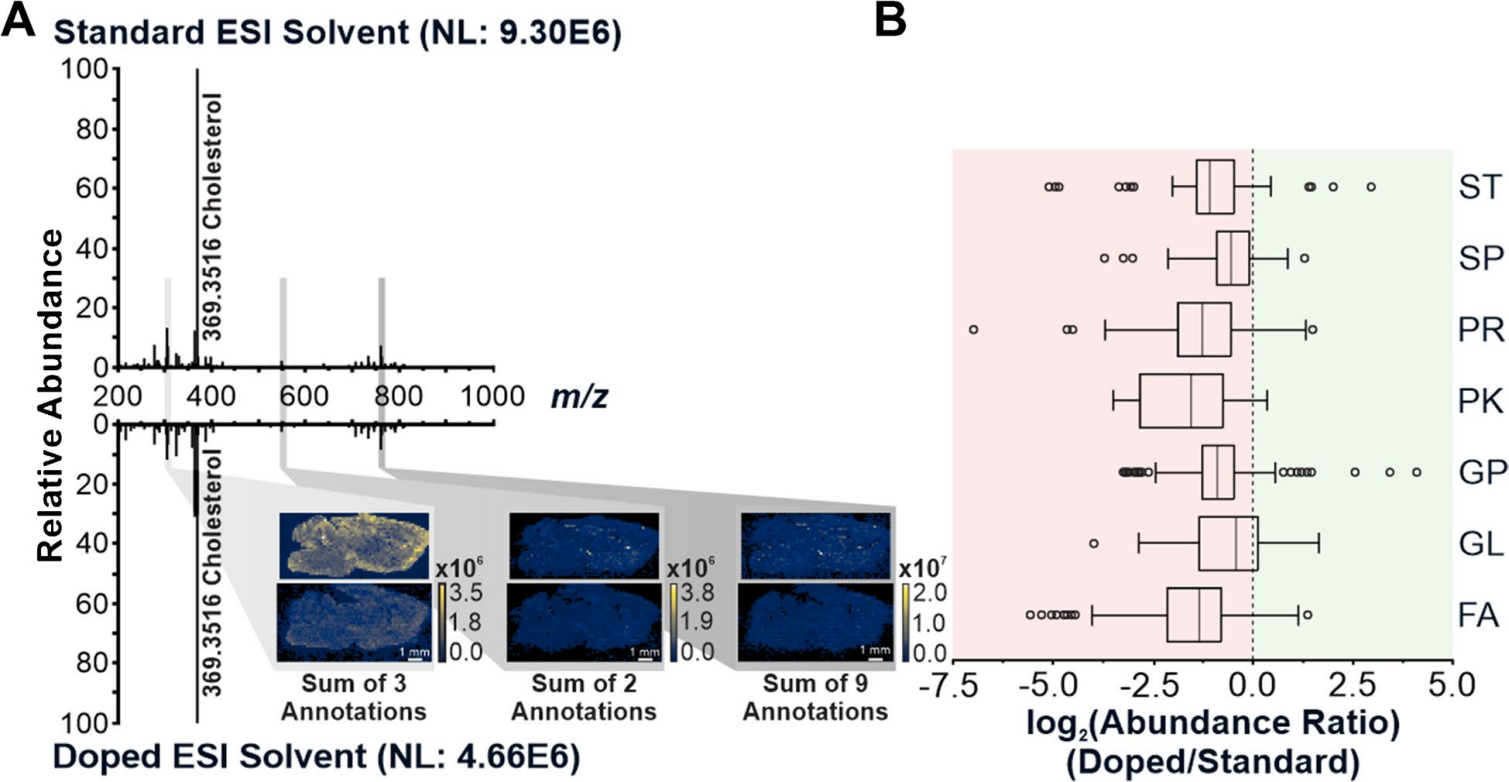
**A** Reflection plot comparing the lipid ion detection between standard and NH_4_F doped ESI solvent with the application of an ice matrix. Summed ion abundance heat map images demonstrate the differences in abundance between the two conditions at the lower, central, and upper mass ranges. The lower (3 annotations: PR01 (*m/z* 305.2475) and FA01 (*m/z* 313.2737, 315.2530)), central (2 annotations: FA07 (*m/z* 548.5401) and SP01 (*m/z* 566.5506)), and upper (9 annotations: GP01 (*m/z* 758.5694, 760.5851, 768.5514, 768.5538, 772.5851), GP01/GP10 (*m/z* 762.6007, 764.5225, 766.5381), and SP01 (*m/z* 761.5886)) mass ranges were represented by lipids with high signal in both tissue conditions and were previously analyzed under alternative conditions [21]. **B** Box plot of the log_2_(abundance ratio) for each lipid separated by the LMSD categorization

The doped solvent has thus shown both comparable and unfavorable detection efficiency for two important lipid analytes. To determine if doping suppressed the detection of lipids overall, a reflection plot comparing the averaged raw mass spectra collected from the ice-treated standard and doped ESI solvent trials was produced (Fig. 8A). The cholesterol peaks showed an almost 2 × increase in abundance, favoring the standard ESI composition. To further demonstrate the differences between the two solvents, three groups of ions were summed to reflect the variance in abundance in the lower (*m/z* 300–325), central (*m/z* 545–570), and upper (*m/z* 745–770) mass ranges. There was a 1.8×, 1.5×, and 1.6 × increase in abundance for the lower, central, and higher mass ranges using standard ESI solvent, respectively (Supplemental Table S1). Of the 14 ions evaluated in these mass groups, 13 (93%) preferred the standard ESI solvent. To further display this preference across all the lipid categories, a box plot depicting the log_2_(doped/standard) abundances was prepared (Fig. 8B).

A mean around zero would represent that, on average, that lipid category had consistent abundances between the standard and doped ESI solvents. Based on Fig. 8B, each of the seven detected lipid categories had very few lipids that had higher abundance with the NH_4_F doping (log_2_(doped/standard) > 0). All lipid types had a mean below zero, affirming that in positive ion mode IR-MALDESI MSI analysis, NH_4_F doping of the ESI solvent hindered detection of lipids.

While it is possible that the formation of other adducts, particularly ammonium adducts, could play a role in the decreased detection at one *m/z*, this does not explain the overall trend. In the case of the four psychosine and cholesterol adducts, after summing together all the adducts, the NH_4_F doped results saw no enhanced detection. It is likely that in addition to the dispersion of analytes across multiple adducts, the highly electronegative fluoride could also interfere with protonation of the lipid species as it draws away the protons to preferentially make HF over [M + H]^+^.

## Conclusions

IR-MALDESI MSI analysis was able to detect and visualize the distribution of the cytotoxic sphingolipid, psychosine, in a sucrose-embedded Twitcher mouse brain model. Colocalization plots revealed an interesting potential relationship between localized psychosine abundance and reduced regional neuronal cholesterol levels. Lipid dysregulation is commonly associated with most neurodegenerative diseases, so, while the abundance of several lipids besides psychosine could be used as biomarkers of GLD, their dysregulation alone could not identify a brain as having GLD specifically. Although effective for previous application in the negative ion mode, ammonium fluoride doping was ineffective at enhancing detection of lipids in positive ion mode IR-MALDESI MSI analysis. While the potential insights observed in this work are encouraging, the limited number of biological replicates reduces statistical power, and further studies with increased replication will be required to establish biological conclusions.

## Supplementary information

Below is the link to the electronic supplementary material.

ESM 1 (DOCX 4.98 MB)

## Data Availability

All data is available through NCSU’s Dryad system: 10.5061/dryad.fttdz096q and through METASPACE: https://metaspace2020.org/project/hunter-2025

## Declarations

### Source of biological material

Twitcher (#000845) mice were purchased from The Jackson Laboratory and bred in-house [57].

### Statement on animal welfare

All mice used in this study were group housed on ventilated racks under standard 12-h light/dark conditions. Mice were fed a 5053 or 5R53 PicoLab Rodent diet with access to water through plumbed Lixit systems, both ad libitum.
