# Supplementary material for: Spatially resolved lipids in a mouse brain model of globoid cell leukodystrophy via IR-MALDESI MSI and parallel reaction monitoring MSI
Source: Anal Bioanal Chem. 2026 Jan 23;418(7):1973–86. doi: 10.1007/s00216-026-06326-3 (PMC12999646; doi:10.1007/s00216-026-06326-3)
Supplement: Supplementary file 1 — (DOCX 4.98 MB) [file 216_2026_6326_MOESM1_ESM.docx]

**Supporting Material**

**Spatially Resolved Lipids in a Mouse Brain Model of Globoid Cell Leukodystrophy via IR-MALDESI MSI and Parallel Reaction Monitoring MSI**

Sierra N. Hunter,^1^ Mary F. Wang,^1^ Brittany N. Thomas,^2,3^ Anthony J. Filiano,^2,3*^ and David C. Muddiman^1^*

*^1^Biological Imaging Laboratory for Disease and Exposure Research (BILDER), Department of Chemistry, North Carolina State University, Raleigh, NC 27695*

*^2^Marcus Center for Cellular Cures, Department of Neurosurgery, Duke University, Durham, NC 27705*

*^3^Department of Pathology, Duke University, Durham, NC 27710*

**Submitted to:** *Analytical and Bioanalytical Chemistry*

**Submitted:** November 17, 2025

**Revised:** December 23, 2025

**Manuscript:** 10 Pages / 5 Figures / 1 Table

**Keywords:** IR-MALDESI; mass spectrometry imaging; Globoid cell leukodystrophy, psychosine

*Authors for Correspondence

Anthony Filiano, PhD

Departments of Neurosurgery, Immunology and Pathology

Marcus Center for Cellular Cures

Duke University

Phone: 919-613-6058

Email: [anthony.filiano@duke.edu](mailto:anthony.filiano@duke.edu)

David C. Muddiman, Ph.D.

Biological Imaging Laboratory for Disease and Exposure Research

Department of Chemistry

North Carolina State University

Phone: 919-513-0084

Email: [dcmuddim@ncsu.edu](mailto:dcmuddim@ncsu.edu)


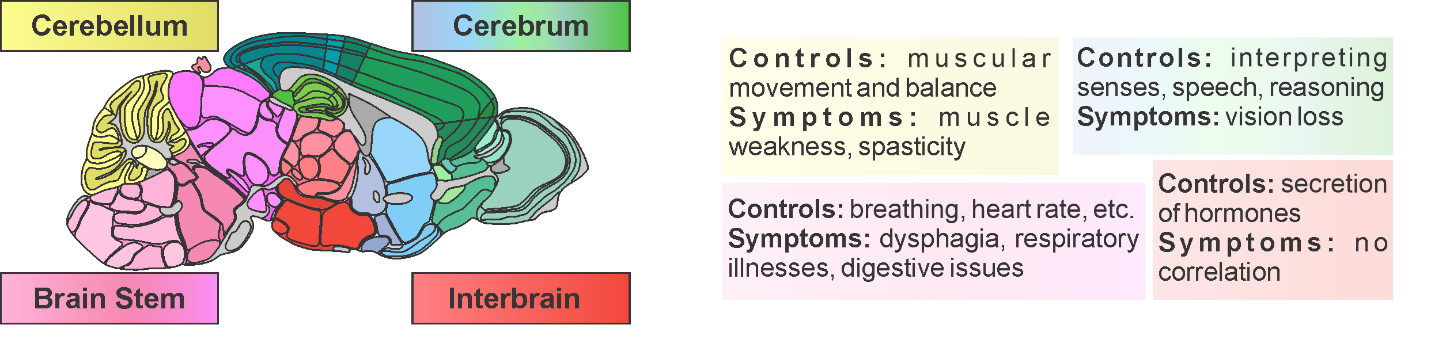


**Figure S1.** Diagram of a mid-sagittal mouse brain section. Four primary regions of interest for GLD are differentiated via color associations. The main bodily functions for each region is reported as well as how that brain region correlates to known and reported symptoms of GLD.


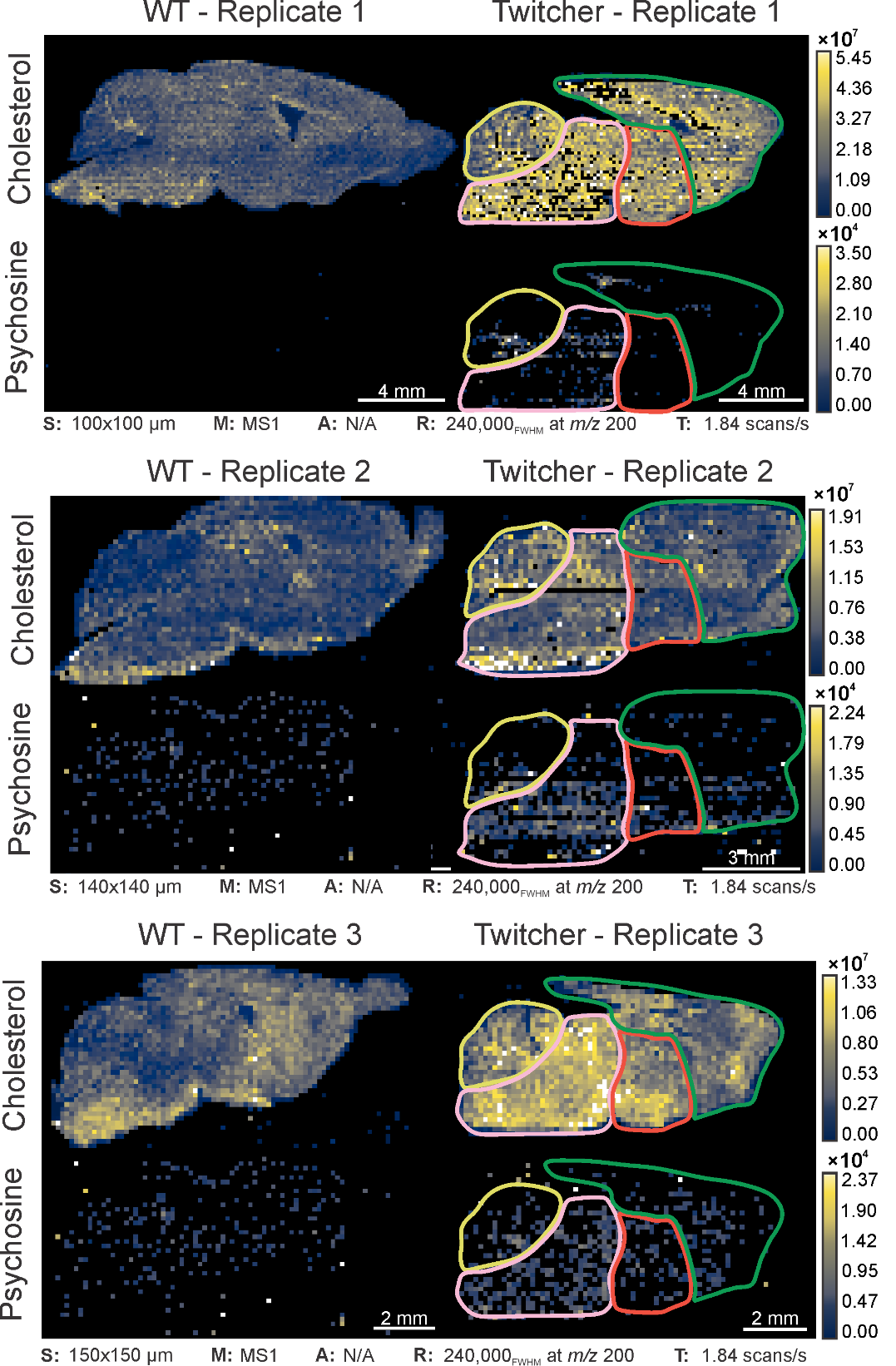

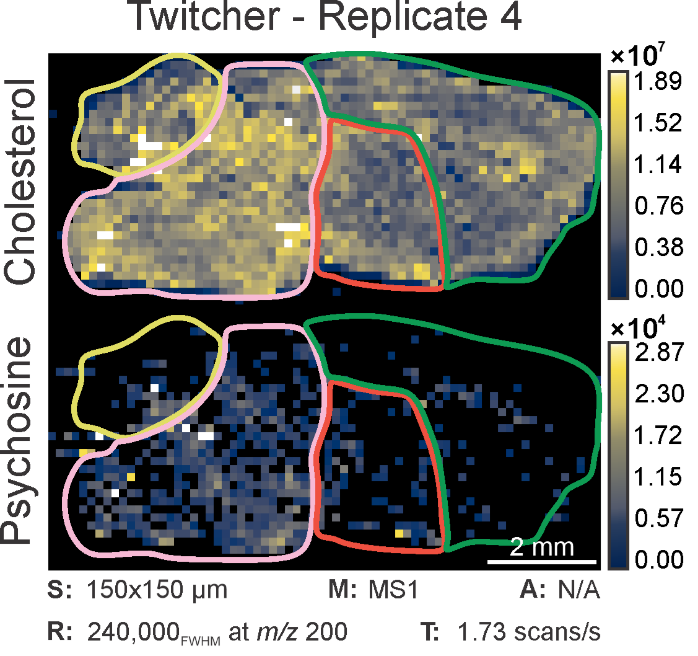


**Figure S2.** Ion heat maps of cholesterol [M + H – H_2_O]^+^ and psychosine [M + H]^+^ *m/z*’s for each replicate used in the IR-MALDESI MSI lipidomic analysis.


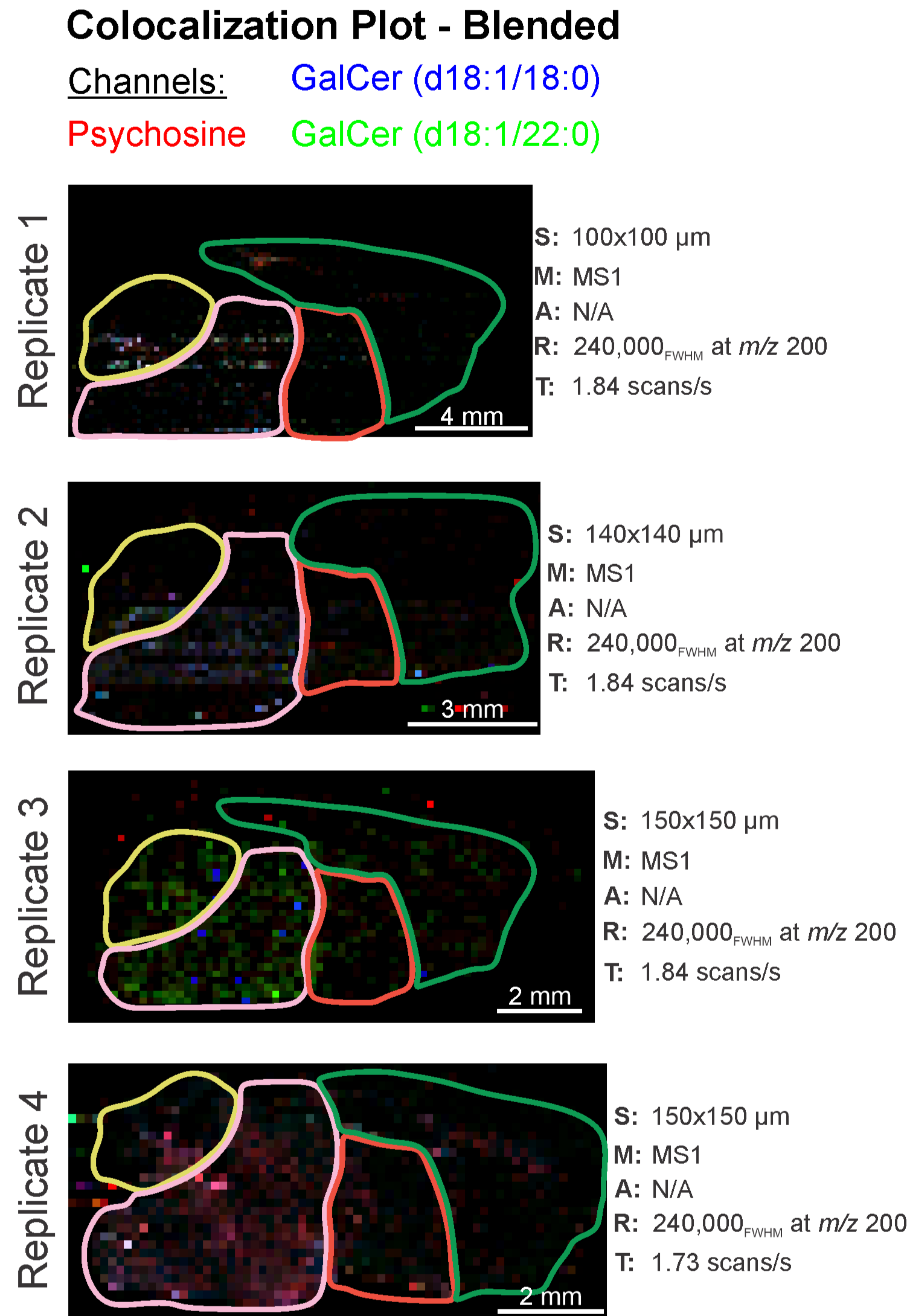


**Figure S3.** Colocalization plot of two GalCer (d18:1) analytes in relation to psychosine for the four Twitcher replicates used in the IR-MALDESI MSI analysis.


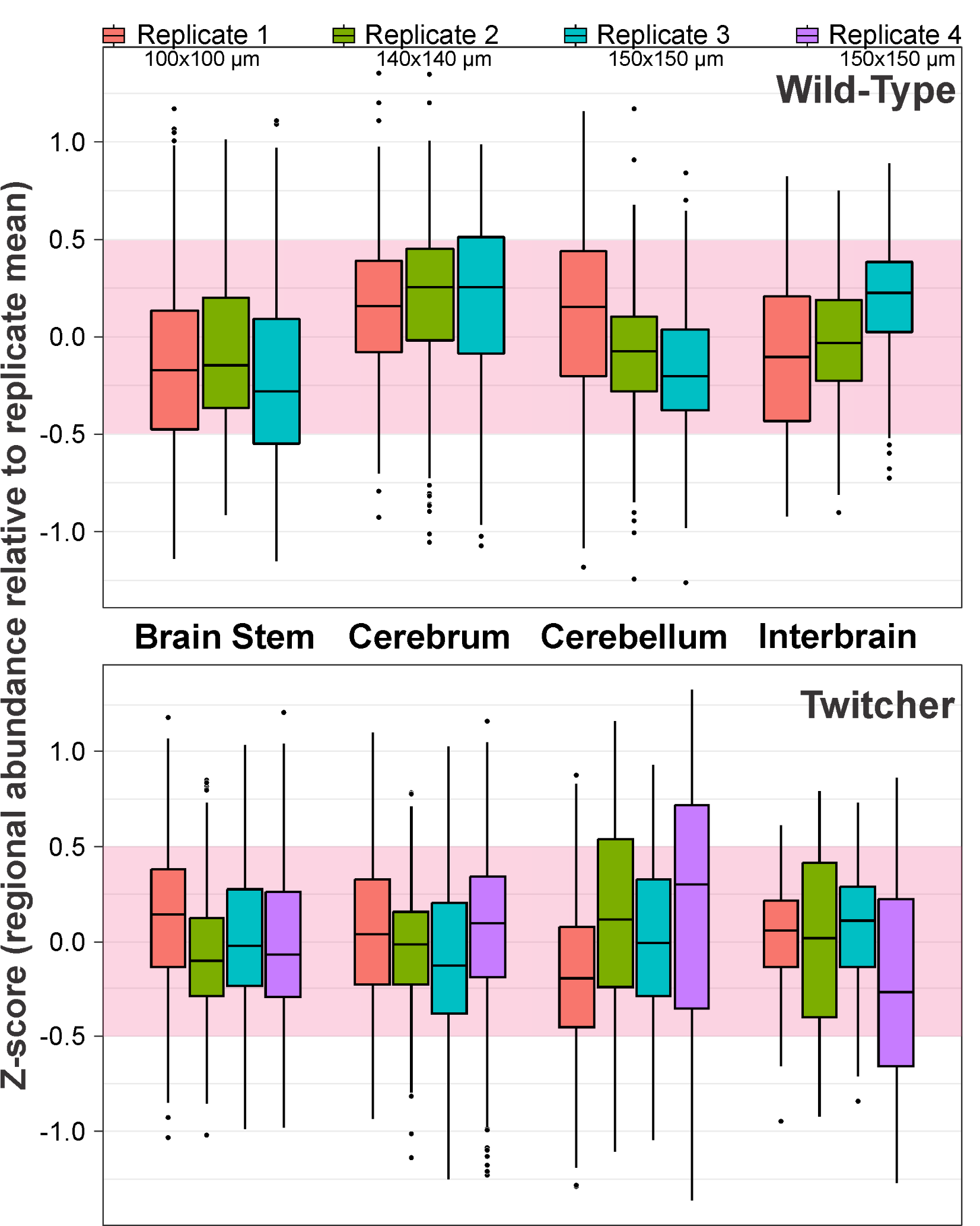


**A**


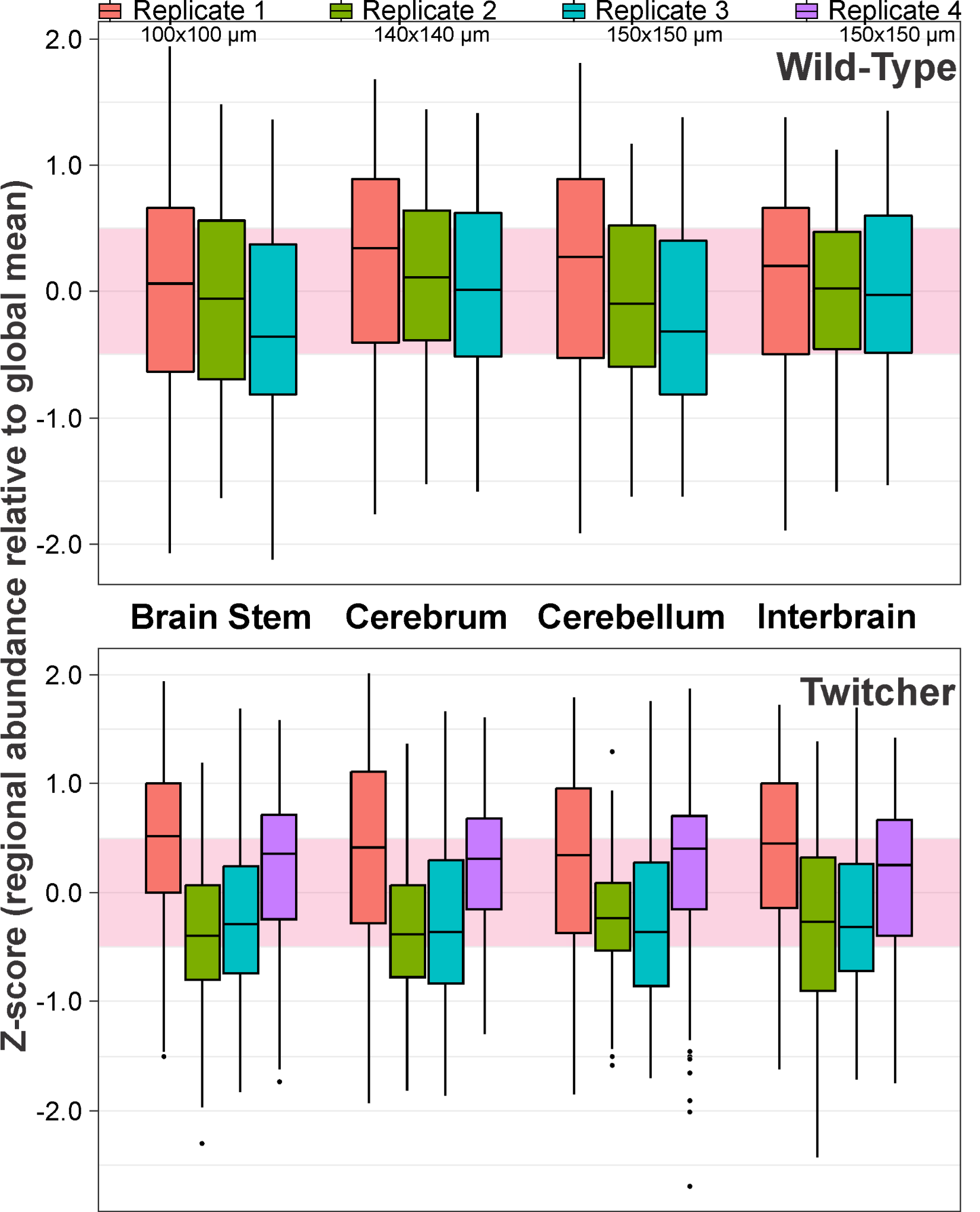


**B**


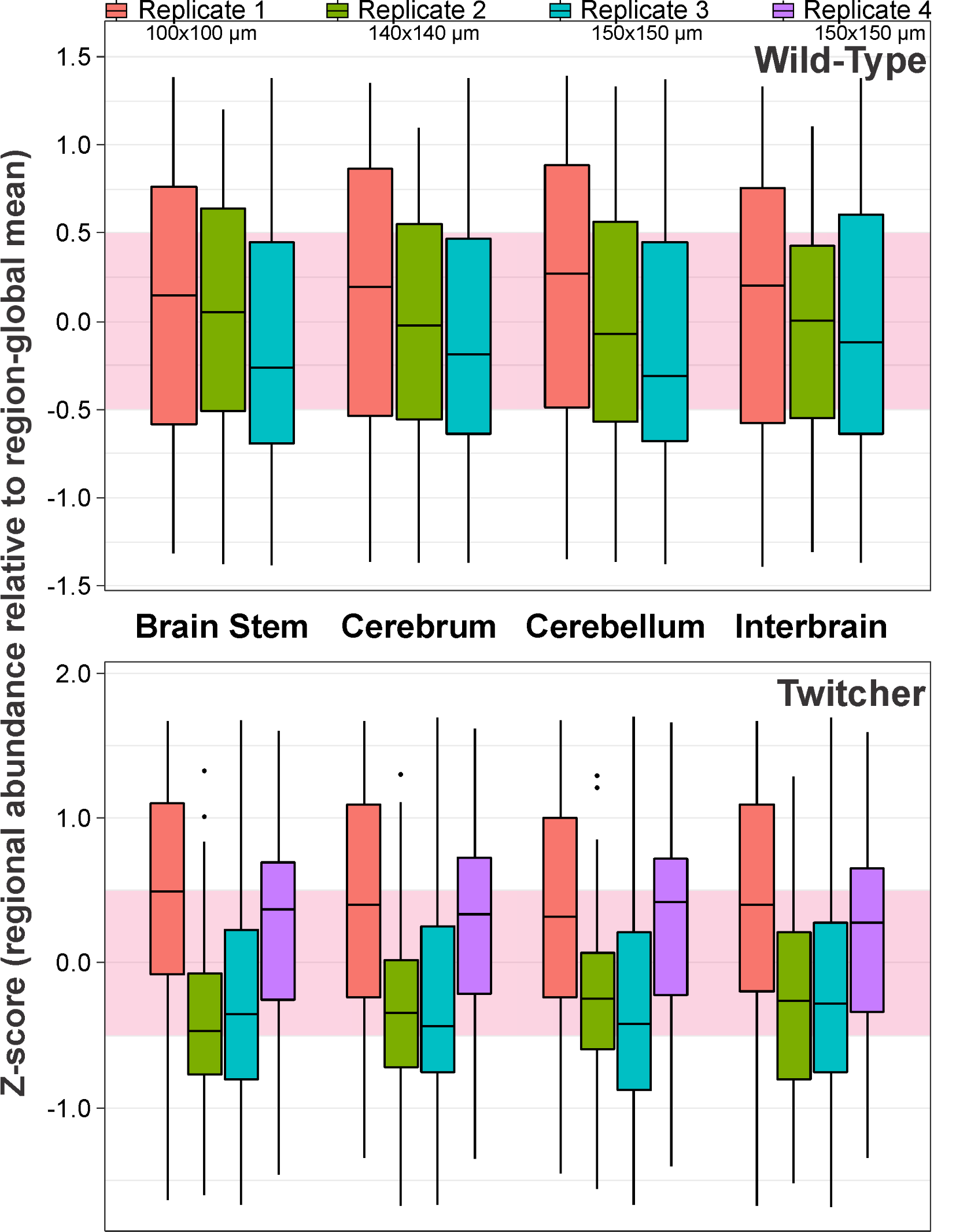


**C**

**Figure S4.** Boxplots of the log-transformed z-scores of analyte abundances across regions of the WT and Twitcher mouse brain replicates. Each z-score was normalized by the **(A)** replicate mean, **(B)** global mean, and **(C)** region-global mean. Replicate means are derived from the average of the bioinformatic ROIs in each individual replicate (n=40). Global means average the bioinformatic ROIs of all replicates of each tissue type (n=120 and 160 for WT and Twitcher respectively). Region-global means average the bioinformatic ROIs of each brain region across the tissue type’s replicates (n=30 and 40 for WT and Twitcher respectively).

**Figure S5.** Volcano plot of the putative on-tissue lipids for the **(A)** cerebellum, **(B)** brain stem, **(C)** cerebrum, and **(D)** interbrain formed by each region’s bioinformatic ROIs across all replicates (n=3 for WT and 4 for Twitcher). Statistical significance follows standard practice where |log_2_fc| >0.6 and -log_10_(pval) greater than -log_10_(0.05) is significant.


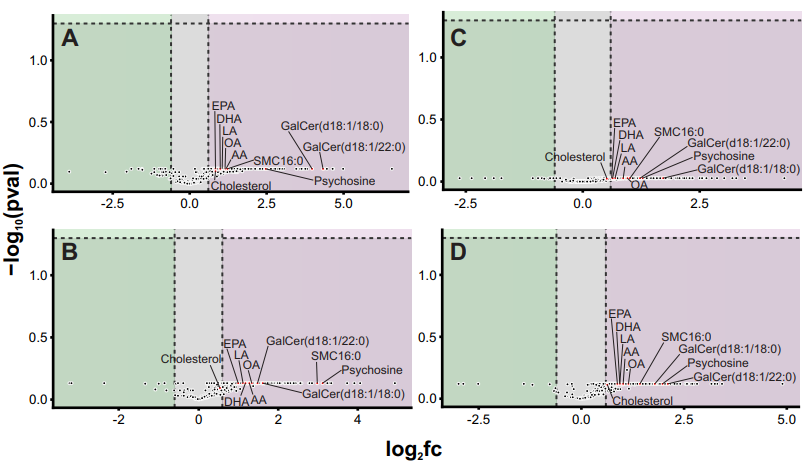


| Positive Ion Mode with Application of Ice Matrices | | | | |
| --- | --- | --- | --- | --- |
| Lower Mass Range (3 Ions) | ESI Solvent | Abundance | Abundance Ratio | |
| PR01 (*m/z* 305.2475) | Standard | 1.40E6 | 2.41 | |
|  | NH_4_F Doped | 5.81E5 |  |  |
| FA01 (*m/z* 313.2737) | Standard | 4.84E4 | 1.91 | |
|  | NH_4_F Doped | 2.53E4 |  |  |
| FA01 (*m/z* 315.2530) | Standard | 6.01E3 | 1.12 | |
|  | NH_4_F Doped | 5.38E3 |  |  |
| Average Abundance Ratio 1.81 | | | | |
| Central Mass Range (2 Ions) | ESI Solvent | Abundance | | Abundance Ratio |
| FA07 (*m/z* 548.5401) | Standard | 2.28E5 | | 2.00 |
|  | NH_4_F Doped | 1.14E5 | |  |
| SP02 (*m/z* 566.5506) | Standard | 1.82E4 | | 1.05 |
|  | NH_4_F Doped | 1.73E4 | |  |
| Average Abundance Ratio 1.53 | | | | |
| Upper Mass Range (9 Ions) | ESI Solvent | Abundance | | Abundance Ratio |
| GP01 (*m/z* 758.5694) | Standard | 5.85E4 | | 1.94 |
|  | NH_4_F Doped | 3.02E4 | |  |
| GP01 (*m/z* 760.5851) | Standard | 7.48E5 | | 1.90 |
|  | NH_4_F Doped | 3.94E5 | |  |
| GP01 (*m/z* 768.5538) | Standard | 1.63E5 | | 1.43 |
|  | NH_4_F Doped | 1.14E5 | |  |
| GP01 (*m/z* 768.5514) | Standard | 3.27E3 | | 0.39 |
|  | NH_4_F Doped | 8.32E3 | |  |
| GP01 (*m/z* 772.5851) | Standard | 4.88E4 | | 2.05 |
|  | NH_4_F Doped | 2.38E4 | |  |
| GP01/GP10 (*m/z* 762.6007) | Standard | 9.25E4 | | 1.82 |
|  | NH_4_F Doped | 5.09E4 | |  |
| GP01/GP10 (*m/z* 764.5225) | Standard | 6.74E4 | | 1.35 |
|  | NH_4_F Doped | 4.98E4 | |  |
| GP01/GP10 (*m/z* 766.5381) | Standard | 3.93E4 | | 1.39 |
|  | NH_4_F Doped | 2.82E4 | |  |
| SP01 (*m/z* 761.5886) | Standard | 3.41E5 | | 1.91 |
|  | NH_4_F Doped | 1.79E5 | |  |
| Average Abundance Ratio 1.58 | | | | |

**Table S1.** Positive ion mode abundance ratio calculations across all ions within each shaded region depicted in **Figure 8A** with averaged ratios for each region (lower, central, and upper mass ranges).
